# HIV risk behaviors among female IDUs in developing and transitional countries

**DOI:** 10.1186/1471-2458-7-271

**Published:** 2007-10-01

**Authors:** Charles M Cleland, Don C Des Jarlais, Theresa E Perlis, Gerry Stimson, Vladimir Poznyak, the WHO Phase II Drug Injection Collaborative Study Group

**Affiliations:** 1 Center for Drug Use and HIV Research, National Development and Research Institutes Inc. 71 West 23rd Street, New York, NY 10010, USA; 2 Baron Edmond de Rothschild Chemical Dependency Institute, Beth Israel Medical Center, 160 Water Street, 24th Floor, New York, NY 10038, USA; 3 International Harm Reduction Association; The Centre for Research on Drugs and Health Behaviour, London School of Hygiene and Tropical Medicine, UK; 4 Department of Mental Health and Substance Dependence, World Health Organization, Geneva, Switzerland; 5 Professor Moruf Adelekan and Dr Rahim Lawal, University of Ilorin Teaching Hospital, Ilorin – Kwara State, Nigeria; Dr Francisco Inacio Bastos, Oswaldo Cruz Foundation, Rio de Janeiro, Brazil; Dr Nguyen Tran Hien and Dr Dao Thi Minh An, Hanoi Medical University, Hanoi, Viet Nam; Dr Sylvia Inchaurraga, Universidad Nacional de Rosario, Rosario, Argentina; Dr Don Des Jarlais and Dr Theresa Perlis, National Development and Research Institutes, Inc., New York, USA; Dr Maristela Monteiro, World Health Organization, Geneva, Switzerland; Prof. V. Navaratnam and B. Vicknasingam, Centre for Drug Research, University Sains Malaysia, Malaysia; Dr Augusto Perez Gomez and Dr Ines Elvira Mejia, Programa RUMBOS, Bogotá, Colombia; Dr Fabio Mesquita, Faculdade de Medicina da USP, Santos, Brazil; Dr Sergey Molochko, Minsk City Narcological Dispensary, Minsk, Belarus; Dr Maurice Odek-Ogunde, United States International University, Nairobi, Kenya; Mr Dimitry Ostrovsky, Foundation Vozvrastcheniye, St. Petersburg, Russia; Dr Vladimir Poznyak, World Health Organization, Geneva, Switzerland; Dr Emran Razzaghi and Dr Afarin Rahimi, Iranian Welfare Organization, Teheran, Iran; Professor Gerry Stimson and Mr Chris Fitch, formerly at Imperial College School of Medicine, London, United Kingdom; Dr Olga Balakireva and Dr Marina Varban, Ukrainian Institute for Social Research, Kiev, Ukraine; Prof. Zunyou Wu and Dr Lorraine Yap, Chinese Academy of Preventive Medicine, Beijing, China

## Abstract

**Background:**

A number of studies suggest females may be more likely to engage in injection and sex risk behavior than males. Most data on gender differences come from industrialized countries, so data are needed in developing countries to determine how well gender differences generalize to these understudied regions.

**Methods:**

Between 1999 and 2003, 2512 male and 672 female current injection drug users (IDUs) were surveyed in ten sites in developing countries around the world (Nairobi, Beijing, Hanoi, Kharkiv, Minsk, St. Petersburg, Bogotá, Gran Rosario, Rio, and Santos). The survey included a variety of questions about demographics, injecting practices and sexual behavior.

**Results:**

Females were more likely to engage in risk behaviors in the context of a sexual relationship with a primary partner while males were more likely to engage in risk behaviors in the context of close friendships and casual sexual relationships. After controlling for injection frequency, and years injecting, these gender differences were fairly consistent across sites.

**Conclusion:**

Gender differences in risk depend on the relational contexts in which risk behaviors occur. The fact that female and male risk behavior often occurs in different relational contexts suggests that different kinds of prevention interventions which are sensitive to these contexts may be necessary.

## Background

Female injection drug users (IDUs) frequently have been found to engage in more risk behavior than males. For example, in a general sample of drug users in Denver, Colorado, Booth [1] found that needle sharing was more common among females than males. Among IDUs in England, receptive sharing of needles and syringes was more common among females than males [2]. Among IDUs not in treatment in Paterson, New Jersey, recent injecting with a sex partner was more common among females [3], and this was often an injection given to the female by the male after the male had already injected. Both receptive and distributive injection equipment sharing were more common among female than male IDUs in Toronto [4].

Females IDUs in Marseille, France were both less likely to clean used needles and less likely to use condoms with a primary sex partner than males [5]. Johnson and colleagues found more needle sharing among female IDUs, and suggested this might have been due to higher depression among females, since females were more depressed and depression was related to needle sharing [6]. Also, data collected as part of the NIDA Cooperative Agreement for AIDS Community-Based Outreach/Intervention Research showed that needle sharing was more frequent among female than male IDUs, and this effect was consistent across the 18 sites studied [7]. There are a number of reasons why female IDUs may engage in more risk behavior. Females may be more stigmatized for their drug use, and stigmatization may lead to more risk behavior. For example, women may avoid using syringe exchanges out of fear that they will be recognized within their community as an injection drug user, leading to eviction or loss of child custody [8].

Although there are plausible reasons for gender differences in injection risk behavior and differences frequently are observed, some studies have failed to find increased risk behavior among female IDUs [9]. For example, Riehman and colleagues [10] found no gender differences in receptive or distributive syringe sharing among California IDUs presenting at syringe exchanges. Also, female and male drug users treated in a New York City emergency department were found to have similar rates of HIV risk behavior [11]. There is also evidence of both more risky and more protective behavior among females; for example, Montgomery and colleagues reported that female IDUs shared needles more frequently, but were more likely to use needle exchange and carry clean syringes than male IDUs [12].

The fact that studies have not always found that female IDUs are more risky may be due in part to males and females engaging in different types of risk behavior [5,13,14]. For example, Bennett and colleagues [2] found that male injectors engaged in more distributive sharing of equipment other than syringes while females receptively shared injection equipment more often. In addition to engaging in different types of risk behavior, female and male IDUs may share syringes and other injection equipment with different types of people. Valente and Vlahov [15] showed that injection risk behavior is often selective, in that it occurs in the context of close personal ties between sexual partners or among a network of injectors. When female IDUs are found to engage in more risk behavior, this may be selective risk behavior that often occurs in the context of a primary sexual relationship [1,3,5,13,16-20]. Female IDUs are more likely to have injecting sex partners than male IDUs [3,17], and this difference may provide more opportunities for female IDUs to selectively engage in injection equipment sharing with those sex partners. This is consistent with findings of a survey of San Francisco IDUs [21], where female IDUs were more likely than males to have borrowed needles and other injection equipment in the past 3 months. Females also were more likely to have been injected by someone else. But it was much more common for female rather than male IDUs to have injected with a sex partner, and females were more likely to borrow needles from a sex partner. Importantly, the relation between gender and both needle sharing and other injection equipment sharing was no longer significant once injecting with a sex partner was controlled. While the relation between female gender and being injected by someone else persisted after controlling for injecting with a sex partner, on the whole the results suggest that a good deal of female injection risk behavior occurs in the context of sexual relationships.

Although most data on gender differences in risk behavior among IDUs come from industrialized countries, a couple of recent studies have reported on gender differences in developing countries. One study conducted in Sichuan Province, China [22] found that female and male IDUs were equally likely to have injected with a used needle from a sex partner in the past year. Receptive needle sharing with friends, sharing other injection equipment, and unprotected sex with primary and casual partners were also found to be similar among female and male IDUs. Differences did emerge on one injection risk behavior, using needles prepared by other people, but male IDUs were more likely than female IDUs to report this behavior. Among young injectors in St. Petersburg, Russia, female IDUs engaged in more sexual risk behavior than male IDUs [23], but it was not clear how much of that difference was due to sex work being more common among females. Also, females and males both shared injection equipment about four and a half times in the past month, with no significant gender difference [24]. However, the females in this study were more likely than the males to report being injected by someone else. The sample in Sichuan was much older than the sample in St. Petersburg and one study asked about risk behavior in the past year while the other asked about the past month; despite these differences, neither study found the expected relation between female gender and the sharing of injection equipment. Thus, these two studies raise the possibility that gender differences that have been found frequently in industrialized countries might not be observed consistently in developing countries.

The purpose of this paper is to examine the consistency of gender effects on injection risk behaviors across multiple sites in developing and transitional countries. Regardless of whether effects are found or not, or suggest males or females are more risky, findings which are consistent across multiple sites are highlighted and contrasted with inconsistent sites.

## Methods

As part of the overall WHO Drug Injection Study Phase II, a cross-sectional seroprevalence and risk behavior survey of injecting drug users was carried out in various cities from mid 1999 through 2003 (see [25] and [26] for a full description of the study). The survey was conducted at different times in each city and data collection typically lasted 6 to 9 months or longer. Centralized survey coordination, including creation of a core questionnaire with additional optional sections, preparation of guidelines for local survey operations, technical support, and compilation of data into a master database, facilitated local questionnaire modification within the core structure, standardized data collection protocols, and uniform database structure. Following the guidelines, each site was required to plan the local survey operations, adapt and translate the prototype questionnaire for local use, hire and train fieldworkers, pilot the questionnaire, identify recruitment locations, develop local sampling/recruitment protocols, select laboratory facilities and arrange for HIV serotesting, recruit and interview subjects, and enter questionnaire data into a computer database. Each participating site assumed responsibility for the precise nature and duration of training which typically lasted between two and five days and covered all aspects of recruitment and questionnaire administration, informed consent procedures, principles of communication with IDUs, safety issues for field workers, record keeping, and data management.

This report covers data collected from male and female injecting drug users (IDUs) in 10 of the 12 cities which participated in the survey including Nairobi (Kenya) in Africa, Beijing (China) and Hanoi (Vietnam) in Asia, Kharkiv (Ukraine), Minsk (Belarus) and St. Petersburg (Russia) in Eastern Europe, and Bogotá (Colombia), Gran Rosario (Argentina), Rio de Janeiro (Brazil) and Santos (Brazil) in South America. In the remaining two cities, Lagos (Nigeria) recruited very few female IDUs, and Penang (Malaysia) recruited none; thus adequate data on female IDUs were unavailable in these cities.

### Ethical review

Principal Investigators (PIs) and the Implementing Agencies were required to adhere to the principles described in the International Guidelines for Ethical Review of Epidemiological Studies [27] as well as to any additional local regulations, and to submit their entire study protocol for review by a local Ethics Board to ensure that potential risks of participant identification or loss of confidentiality were minimized [25]. Each site was required to prepare an informed consent information sheet clarifying possible implications of study participation, to show (or read) to participants, and to obtain written or verbal consent from participants.

### Confidentiality

In accordance with guidelines of the Department of Mental Health and Substance Dependence, WHO (WHO/MSB), potential risks to individual study recruits (e.g., risk of violence, breach of law, loss of confidentiality, etc.) and procedures for minimizing such risks were addressed in the initial proposals submitted to WHO/MSB by individual site PIs and Implementing Agencies. In addition the topic was covered in the Survey Operations Manual and also fully discussed during a 4-day planning meeting in St. Petersburg, Russia attended by the WHO/MSB project team, the Coordinating Center teams, and representatives from all participating cities except Nairobi (which had not yet been selected at that time). Confidentiality issues covered the recruitment process, privacy during questionnaire administration, data storage, and linking of laboratory data for the purposes of giving out test results. However, since human subjects protection issues varied across sites, the specific details of procedures for minimizing such risks were determined by individual PIs with review and approval by local ethical review boards. Moreover, each participating site assumed responsibility for the precise nature and duration of field worker training including confidentiality issues. Data sets submitted to the NYC Survey Coordinating Center (NYC-SCC) for analysis did not include any names.

### Eligibility for survey enrollment

Recruitment of IDUs was restricted to those who had injected during the 2 months prior to enrollment. Subjects were recruited through community outreach in all cities, and from drug treatment entrants in Asian and Eastern European cities. Since most of the questionnaire sections on drug use and risk behaviors covered the 6-month period preceding study enrollment, recruitment from treatment settings was limited to clients newly-admitted to a treatment program (within the past 30 days), since such clients could be considered to have been 'part of the community' for most of the six-month period. Apart from the current course of treatment, the subject should not have been in that same treatment program or any other treatment program during the preceding 6 months. Community recruits were drawn from any non-treatment settings, preferably from street locations. If a person recruited from a community setting was currently attending drug treatment this did not render him/her ineligible as a community recruit.

### Sampling procedures and recruitment of subjects

Owing to the vastly different situations across cities, a completely uniform approach to sampling and recruitment was not considered feasible. Overall guidelines were provided, but the ultimate decision on design and implementation of local sampling and recruitment protocols was made by each site investigator in consultation with the survey coordinators. For treatment recruitment, where random selection was not feasible (due primarily to small numbers of incoming patients), sequential new admits were screened for eligibility and all meeting the criteria were invited to participate in the survey. Since random selection of subjects in the community was typically not feasible, techniques such as peer-referral, snowball sampling, and targeted sampling, were used in order to access the hidden populations. Investigators were asked to make every effort to obtain a diverse sample of the non-treatment population in the area.

Persons recruited from drug treatment facilities were usually interviewed in a specially assigned room inside the facility where recruitment took place. On the other hand, community recruits were typically recruited in one location but interviewed in private rooms elsewhere. The variety of interviewing venues included rooms in lodging houses or a church hall in Nairobi, confidential locations chosen by participants in Beijing, commune health centers in Hanoi, the Regional AIDS Centre in Kharkiv, the Narcological Dispensary, National Center for AIDS Prevention or the Syringe Exchange Outlet in Minsk, a bus (small van) which traveled to selected locations in St. Petersburg, and private offices in cooperating NGOs in Bogotá. Locations were often chosen to minimize visibility to citizens and police or even to protect the field staff from violence from other drug users. In some cases interviews had to be conducted in more public places such as cafes, discos, etc. but every attempt was made to maintain confidentiality.

In Beijing and Nairobi, monetary incentives were offered for survey participation, and in Hanoi, Bogotá and Rio de Janeiro participants' transportation expenses were paid by the project. Additionally, in Beijing, Hanoi and Bogotá, incentives were given to recruits to refer other drug users to the study. For the community sample, rates of refusal to participate in the study were reported by only three of the sites. In Kharkiv 3%–5% of eligible recruits refused to be interviewed. Reasons include anxiety about being identified or "registered" as a drug addict, no interest in the study, or poor state of health. The 9% refusal rate in St. Petersburg appeared to be primarily due to the length of the questionnaire. In Bogotá, 30% of persons contacted did not keep their appointments, and although specific reasons for refusal were not always available, some of the drug users reported fear of receiving a positive blood test result, dread of the phlebotomy procedure, or apprehension of identification during the procedure. Additionally, the Nairobi team reported that the refusal rate appeared to be "very low", mostly from persons who felt that the monetary incentive was insufficient, and the Minsk team reported that most of the approached IDUs agreed to participate in the study. No information was received for the remaining sites. Although specific refusal rates among drug treatment entrants were not reported by any of the sites, the overall impression was that persons in drug treatment were less likely to refuse because they were already openly identified as drug users. In particular, the Minsk report indicated that participation in the study provided a welcome change in the everyday routine activities in the treatment center.

### Questionnaire

Although sites were permitted some flexibility to include questions of local interest only, the core part of the structured questionnaire comprised questions to be asked at all study sites.

Translation of the questionnaire was carried out locally at each site – into Mandarin in Beijing, Vietnamese in Hanoi, Spanish in Bogotá and Gran Rosario, and Portuguese in Rio de Janeiro and Santos. The three Eastern European countries collaborated on translation into Russian. Unfortunately, limited resources precluded back-translation. In Nairobi, the English version was used. The locally-adapted structured questionnaire was administered by a trained interviewer to each eligible recruit.

Only two sites reported the questionnaire administration time. In Beijing it was about 30 minutes, whereas Minsk reported between 1 hour 30 minutes and 1 hour 50 minutes. However, Beijing had shortened the local version of the questionnaire based on rapid assessment findings and used almost none of the optional sections whereas the Minsk questionnaire included some of the longer local option sections. Both the Kharkiv and St. Petersburg teams indicated that the questionnaire appeared overly long. Survey questions were necessarily translated for administration at each study site, and the description of measures below presents the English versions of each question.

### Measures

#### Daily injection

IDUs were asked how often during the past 6 months they injected speedball, heroin alone, cocaine alone, methamphetamines, or any other substance injected locally. Possible responses included the following: "Never"; "Less than once a month"; "1 to 3 times a month"; "About once a week"; "2 to 3 times a week"; "4 to 6 times a week"; "About once a day"; "2–3 times a day, almost every day"; and "4 or more times a day, almost every day". If daily or more frequent injecting was indicated for any injected substance, then the IDU was considered a daily injector.

#### Years injecting

This was determined by subtracting age of first injection from age at interview. This difference was then subjected to a natural logarithm transformation for generalized linear models.

#### Sex work

IDUs were asked, "In the last 6 months how often have you had a client who gave you money or goods for sex?" and "In the last 6 months how often have you had a client who gave you drugs for sex?" If responses indicated sex work for money, goods, or drugs with any frequency, then the IDU was considered a sex worker. Male IDUs in the Hanoi site were not asked these two questions, so sex worker status was missing.

#### Risk behaviors

Survey items asked about the following twelve risk behaviors: receptive syringe sharing (i.e., receiving a used needle/syringe from another IDU); distributive syringe sharing (i.e., passing on a used needle/syringe to another IDU); cooker/cotton/rinse water sharing; drawing from a common drug solution; injecting with a pre-filled syringe; frontloading/backloading/splitting; receptive sharing with a primary sex partner; receptive sharing with a close friend; distributive sharing with a primary sex partner; distributive sharing with a close friend; unprotected sex with a primary sex partner; unprotected sex with a casual sex partner. IDUs were asked how often in the last 6 months they had engaged in each risk behavior, and these frequencies were then recoded to indicate simply whether or not that the behavior occurred. IDUs in Beijing were not asked about distributive sharing with primary sex partners and with close friends, so this site could not be included in any analyses of those two risk behaviors.

### Analysis

Site-specific bivariate analyses of demographic and other characteristics were undertaken to better understand gender differences. Differences between males and females were tested for significance with t-tests for continuous variables and Chi-squared tests for categorical variables.

Each of the twelve risk behaviors was examined separately. As a first step, site-specific logistic regression models were fit to determine the effect of female gender on a specific risk behavior within each site. Daily injection and years injecting were included as covariates in each of the site-specific models because bivariate analyses showed that daily injection was significantly related to all twelve of the risk behaviors and years injecting was significantly related to ten of the risk behaviors.

Next, multi-site models were used to assess the consistency of gender effects across sites, and to identify individual sites in which the gender effect differed from other sites in size or direction. The initial models were fit with main effects for site, gender, daily injection, and years injecting, and two-way interactions between site and each of the other three main effect variables. For each behavior, the initial model was simplified by removing two-way interaction terms if that removal improved the Akaike Information Criterion (AIC [28]). If the site by gender interaction effect was retained, this indicated that gender effects were inconsistent across sites, while removal of the site by gender interaction effect indicated that any apparent inconsistencies across sites were merely due to sampling variation. If the site by gender interaction term was removed, the resulting multi-site model provided a single overall estimate of the gender effect across sites controlling for differences in site, daily injection, and years injecting.

**Figure 1:**
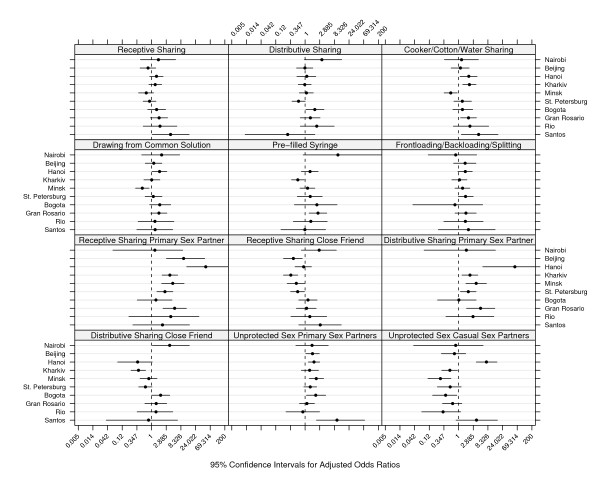
**Adjusted Effect of Female Gender on Injection Risk**. 95% confidence intervals for adjusted odds ratios by site and type of risk.

For risk behaviors in which a site by gender interaction effect remained, we determined the most inconsistent site by examination of the adjusted odds ratios in Figure 1, then re-applied the AIC criterion to a model without the inconsistent site. This process was repeated until we arrived at a model with no site by gender interaction effect to provide an estimate of the overall gender effect for the set of consistent sites. Adjusted odds ratios for the inconsistent or outlying sites were obtained from the site-specific models described earlier.

Statistical significance was evaluated using 2-sided tests and an alpha level of .05 throughout.

## Results

Gender differences within each site are presented for demographics and other key variables (Table 1). Overall, females tended to be slightly younger than males, to have similar levels of education, and were more likely to be married. However, in Hanoi and Nairobi, where the majority of female IDUs were sex workers, females were less likely to be married than males. Sex work was far more common among females than males and at least 25% of females in Nairobi, Hanoi, St. Petersburg, Rio de Janeiro and Santos reported sex with clients. Females were less likely to derive their main income from legal employment. Overall, female IDUs were more likely to be new injectors with shorter injection careers. Apart from a couple of isolated cases, heroin or cocaine use did not differ by gender, but IDUs in south American cities clearly favored cocaine, whereas heroin was the primary injection drug in most of the remaining cities. Gender differences on injection frequency were inconsistent, with daily injection occurring more among females in Hanoi, St. Petersburg, and Santos but more among males in Kharkiv. However, it is notable that injection frequency in the "cocaine" sites was considerably lower than elsewhere for both males and females.

**Table 1:** Demographic and injection drug use characteristics

				Age		12 or More Years Education		Currently Married		Any Sex with Clients		Main Source of Income Legal Work	
		Female N	Male N	Female Mean (SD)	Male Mean (SD)	Female %	Male %	Female %	Male %	Female %	Male %	Female %	Male %
Africa	Nairobi	14	92	28 (8)	29 (7)	7	25	0	23*	86	2*	21	67*
Asia	Beijing	88	278	28 (6)	31 (7)*	26	10*	47	38	3	<1*	26	59*
	Hanoi	119	526	25 (5)	29 (7)*	20	24	11	29*	77	-	13	63*
Eastern	Kharkiv	112	326	24 (5)	25 (6)	38	28*	35	28	7	<1*	59	64
Europe	Minsk	91	309	23 (5)	24 (5)*	62	51	37	23*	3	1	36	57*
	St. Petersburg	103	297	23 (5)	24 (4)	45	45	34	24*	25	<1*	35	57*
South	Bogota	49	188	23 (8)	23 (6)	20	22	29	13*	8	1*	12	27*
America	Gran Rosario	68	261	29 (8)	28 (8)	18	10	48	34*	19	3*	43	43
	Rio de Janeiro	12	177	29 (10)	29 (10)	25	14	42	24	25	17	33	77*
	Santos	16	58	30 (7)	33 (9)	0	2	38	28	44	14*	31	72*
Africa	Nairobi	14	92	5 (4)	6 (3)	57	27*	93	92	100	99	0	2
Asia	Beijing	88	278	2 (2)	2 (3)	81	74	95	92	93	96	0	1
	Hanoi	119	526	2 (3)	3 (4)*	76	70	97	83*	94	95	-	-
Eastern	Kharkiv	112	326	5 (4)	6 (5)	46	35*	50	70*	9	21*	4	4
Europe	Minsk	91	309	4 (3)	5 (4)*	53	46	57	59	79	86	10	8
	St. Petersburg	103	297	5 (5)	5 (4)	45	37	92	84*	94	92	2	4
South	Bogota	49	188	4 (5)	5 (5)	63	60	4	2	14	22	14	51*
America	Gran Rosario	68	261	11 (7)	10 (7)	13	22	15	15	1	2	97	93
	Rio de Janeiro	12	177	9 (9)	10 (9)	50	25	8	5	8	3	100	98
	Santos	16	58	9 (8)	12 (8)	19	12	44	19	6	3	100	100

**Notes**: * *p *< .05

Table 2 presents summaries of risk behaviors by site. It is important to note the considerable variability in these risk behaviors across study sites. For example, only 19% of IDUs in Hanoi had engaged in receptive syringe sharing in the past 6 months, while more than half (54%) of St. Petersburg IDUs did so. These site differences are taken into account when examining gender differences in the multisite models described below. The consistency of any gender differences across sites also is a focus given the variability across sites in the level of each risk behavior.

**Table 2:** Risk behaviors by site

	Nairobi (n = 106)	Beijing (n = 366)	Hanoi (n = 645)	Kharkiv (n = 438)	Minsk (n = 400)	St. Petersburg (n = 400)	Bogota (n = 237)	Gran Rosario (n = 329)	Rio de Janeiro (n = 189)	Santos (n = 74)
Receptive Sharing	28	27	19	52	30	54	47	23	36	22
Distributive Sharing	44	22	9	47	32	65	44	16	39	15
Cooker/Cotton/RinseWater Sharing	40	16	9	28	59	81	68	30	48	43
Drawing From a Common Solution	18	20	14	82	68	80	67	27	63	32
Pre-Filled Syringe	3	1	11	61	26	6	3	20	32	18
Frontloading/Backloading/Splitting	14	9	14	23	25	69	2	13	17	8
Receptive Sharing Primary Sex Partner	6	3	3	16	7	15	6	8	3	7
Receptive Sharing Close Friend	22	23	14	39	23	36	34	18	26	15
Distributive Sharing Primary Sex Partner	5	-	1	16	8	17	5	5	8	3
Distributive Sharing Close Friend	42	-	5	37	20	40	37	13	26	8
Unprotected Sex Primary Partners	39	47	40	83	58	38	58	50	53	51
Unprotected Sex Casual Partners	7	10	5	27	20	12	27	25	50	18

**Notes**: Cell contents are the percentage of IDUs engaging in the risk behavior in the past 6 months. IDUs in Beijing were not asked about distributive sharing with primary sex partners and close friends. Missing data for other sites and behaviors ranged from 0–10% but was less than 1% in 75% of the 118 cells above.

Figure 1 presents 95% confidence intervals around adjusted odds ratios describing the effect of female gender on each risk behavior within each site. The figure was constructed on the logit scale, but to facilitate interpretation of female gender effect sizes, values were labeled with the corresponding odds ratio. Each of these odds ratios are adjusted for the effects of daily injection, and years injecting. There were problems fitting these site-specific models in two cases. In Beijing, only two IDUs injected with a pre-filled syringe, and these were both males. In Santos, only two IDUs engaged in distributive syringe sharing with a primary sex partner, and these were both males. In both of these cases, the odds ratio estimated for the adjusted gender effect was very extreme and the standard error for the gender coefficient also was quite large. Therefore, the effects in these two cases were dropped from Figure 1. Also, for the relevant risk behaviors, these sites were not included in the multi-site models described below.

In the multisite analyses, for 6 of the 12 risk behaviors considered there was no evidence of differential gender effects across sites (Table 3). Overall, females were significantly more likely to engage in frontloading/backloading/splitting, distributive syringe sharing with a primary sex partner, and unprotected sex with a primary sex partner. On the other hand, there were no significant gender effects on receptive syringe sharing, distributive syringe sharing, and drawing from a common drug solution.

**Table 3:** Adjusted effects of gender on risk behavior

	Consistent Sites	Inconsistent/outlying Sites	Data not available
Receptive Sharing	1.14 (0.94 – 1.37)	-	-
Distributive Sharing	1.06 (0.87 – 1.30)	-	-
Cooker/Cotton/Rinse Water Sharing	1.79 (1.43 – 2.24)	Minsk: 0.57 (0.35 – 0.91)	-
Drawing From a Common Solution	1.15 (0.93 – 1.42)	-	-
Pre-Filled Syringe	1.58 (1.18 – 2.10)	Kharkiv: 0.59 (0.38 – 0.93)	Beijing
Frontloading/Backloading/Splitting	1.48 (1.17 – 1.85)	-	-
Receptive Sharing Primary Sex Partner	3.67 (2.74 – 4.91)	Hanoi: 50.23 (12.67 – 351.20)	-
Receptive Sharing Close Friend	0.78 (0.61 – 0.98)	Kharkiv: 0.36 (0.21 – 0.59)	-
Distributive Sharing Primary Sex Partner	2.62 (1.94 – 3.54)	-	Beijing and Santos
Distributive Sharing Close Friend	0.63 (0.48 – 0.82)	Nairobi: 3.70 (1.06 – 15.36)	Beijing
		Bogota: 1.92 (1.00 – 3.70)	
Unprotected Sex Primary Partners	1.67 (1.39 – 2.00)	-	-
Unprotected Sex Casual Partners	0.52 (0.39 – 0.69)	Hanoi: 7.48 (3.61 – 16.01)	-

**Notes**: Cell contents are adjusted odds ratios with 95% profile likelihood confidence intervals in parentheses. In Beijing, only two IDUs injected with a pre-filled syringe, and these were both males. In Santos, only 2 IDUs engaged in distributive syringe sharing with a primary sex partner, and these were both males. In both of these cases, the odds ratio estimated for the adjusted gender effect was very extreme and the standard error for the gender coefficient also was quite large. IDUs in Beijing were not asked about distributive sharing with primary sex partners and close friends.

For each of the remaining six risk behaviors, the gender effect in one or more sites diverged significantly from the other sites. Across all sites except for Minsk females were significantly more likely to engage in cooker, cotton, and rinse water sharing, whereas in Minsk females were significantly less likely to engage in this risk behavior. With the exception of IDUs in Kharkiv females were more likely to inject with a prefilled-syringe, but in Kharkiv the opposite was true. Although females were significantly more likely to engage in receptive syringe sharing with a primary sex partner in all sites, the gender effect was significantly stronger in Hanoi than the other sites. On the other hand, while females were significantly less likely to engage in receptive syringe sharing with a close friend across all sites, the gender difference was larger in Kharkiv than other sites. Across seven of the sites females were significantly less likely to engage in distributive syringe sharing with a close friend, but in Nairobi and Bogotá females were more likely to engage in this risk behavior. Finally, across all sites except Hanoi, females were significantly less likely to have unprotected sex with a casual partner, but this was not true in Hanoi where females were more likely to engage in the behavior.

### Exploring the role of sex work

Female IDUs engage in sex work, often to support drug use. Some have found sex work in females IDUs associated with more risk behavior [29,30] while others have not [31]. Because apparent gender differences may be due only to the fact that females are more likely to engage in sex work, it is important to consider sex work when estimating gender differences. As Table 1 shows, sex workers of both genders were not consistently sampled across sites. While this substantially limits our ability to control for sex work, we tried to determine whether any of the gender effects would change or need to be qualified if sex work were included either as an additional covariate where this was possible. When sex work was included as an additional covariate in the site-specific models described above, the direction and significance of gender effects were essentially the same. Another approach was to confirm that the gender effects reported above were observed among IDUs not engaging in sex work. This was found to be the case, as gender effects in the total sample were also very similar in pattern to gender effects within the subgroup of IDUs who had not engaged in sex work.

## Discussion

### Summary of findings

A relatively consistent pattern of gender differences emerged. Females were more likely than males to engage in injection and sex risk behavior with primary sex partners. Males were more likely than females to engage in injection risk behavior with close friends and to engage in sex risk behavior with casual partners, and this was largely consistent across sites. There was a consistent lack of gender differences on several risk variables that did not specify the relational context of the risk behavior (receptive syringe sharing, distributive syringe sharing, and drawing from a common drug solution).

Overall, gender differences were found when the relational context of the risk behavior was specified, but males and females were similar when the questions asked about risk in general and no context was specified. There were some exceptions to this overall pattern. Females were consistently more likely to frontload, backload or split. In sites other than Kharkiv, females were more likely to inject with a pre-filled syringe. Also, females in sites other than Minsk were more likely to share cookers, cotton, or rinse water. In each of these cases, gender differences were smaller in size than for the behaviors for which relational context was specified.

Female IDUs were much more likely to engage in sex work. When sex work was added as a covariate, this did not change the basic pattern of gender differences. Also, the pattern of gender effects observed in the total sample was quite consistent with the pattern observed only among IDUs not engaging in sex work. This suggests that the current pattern of gender effects may generalize fairly well to other samples of IDUs who are not engaged in sex work. Because sex workers of both genders were not consistently sampled across the sites, the current sample's ability to address gender effects among sex workers is far more limited.

### Implications

Prevention programs which take into account the interpersonal/sexual relationships between female and male IDUs are needed. There are a number of ways this could be done. One approach is to highlight sexual relationships as a salient context for injection risk behavior and emphasize the avoidance of syringe sharing and other risk behaviors with sexual partners. But it is not clear how feasible that would be, particularly for IDUs in sexual relationships of longer duration. For couples who find it very difficult to avoid sharing injection equipment, a better prevention strategy might be to try to isolate the couple so that neither partner engages in risk behavior outside of the couple. Valente and Vlahov [15] noted the high rate of turnover within IDU networks. Our current understanding of the nature and duration of IDU sexual relationships is very limited, and a better understanding is critical to developing optimal and feasible approaches to prevention. Prevention messages which focus on containing risk behavior within a couple would be more effective if IDU sexual relationships are longer-lasting but less effective if these relationships are typically serially monogamous but short-lived. These characteristics of IDU sexual relationships may vary across contexts even though gender effects are somewhat consistent.

There is a need for prevention programs to reach and work with female IDUs. The drug and sex networks of females may overlap more substantially than for males, possibly making risk reduction more difficult [17].

### Limitations

In several of the study sites, it was difficult to recruit female IDUs for a variety of reasons [25], and several sites remarked on the difficulty of recruiting female IDUs who tended to be a more "hidden" group than male IDUs. Even in cities where "official" data on drug users were available there was no breakdown by mode of administration, thus it is not possible to obtain an accurate assessment of the extent to which female IDUs were undersampled. It also is not entirely clear how representative the sample in each site is of all male and female current IDUs in those sites. In a few sites, females tended to be younger, and this might partly account for differences in risk. However, given that most respondents were in their 20s or 30s, the size of the age differences between males and females in each site did not seem large enough to have an impact on injection risk behavior. There was substantial variation in the proportion of females included in the sample across sites. In sites with a small number of female IDUs (Nairobi, Rio, and Santos), there is a great deal more uncertainty in the size of gender differences than in sites with a substantial number of females. There was also substantial variation in the proportion of females engaging in sex work across cities. In sites with few or no sex workers in the sample, there is a great deal of uncertainty in the effect of sex work on risk, and that partly carries over to uncertainty about gender differences.

The proportion of females recruited in each site was not necessarily reflective of the population proportion, and was based largely on specific sampling approaches in each site. For example, in Hanoi, recruitment was undertaken at a rehabilitation center for female sex workers (FSW) since it was known that there was a high prevalence of IDU among FSW. In other sites such as Kharkiv and Minsk, the sample proportion of females is consistent with local estimates of the proportion of female IDUs in the population. In the limited information on refusal to participate available from five of the ten sites, there was no indication of differential refusal by gender.

Finally, all data were obtained by self-report, which may be subject to poor memory or deception. Also, the data come from straightforward survey items with excellent face validity, but no other information on the validity or reliability of the items has been presented.

## Conclusion

Surveys of male and female IDUs in additional locations are needed to continue to explore the degree to which gender differences generalize across a variety of regions and local circumstances. When investigating gender differences in risk, it is important to ask about the context of the behavior in addition to the amount. It is important to control for other factors related to risk behavior in order to isolate the effect of gender. More work is needed to understand the duration and other features of the sexual relationships of IDUs. In particular, it would be useful to investigate how relationship features such as duration, exclusivity, and embeddedness in wider IDU networks are related to injection risk behavior.

## Competing interests

The author(s) declare that they have no competing interests.

## Authors' contributions

DDJ and TP coordinated the questionnaire and seroprevalence survey component of the study and provided technical support to participating sites. GS directed coordination and technical support for the rapid assessment component of the study. VP had overall responsibility for contractual arrangements with individual sites in accordance with the procedures of the WHO, and for study implementation. CMC, TP, and DDJ prepared the article. All of the authors contributed to the final version of the article.

## Pre-publication history

The pre-publication history for this paper can be accessed here:


